# Susceptibility of *Pimephales promelas* and *Carassius auratus* to a strain of koi herpesvirus isolated from wild *Cyprinus carpio* in North America

**DOI:** 10.1038/s41598-021-81477-0

**Published:** 2021-01-21

**Authors:** Isaiah E. Tolo, Soumesh K. Padhi, Keiffer Williams, Vikash Singh, Sophie Halvorson, Sunil K. Mor, Nicholas B. D. Phelps

**Affiliations:** 1 Minnesota Aquatic Invasive Species Research Center and the Department of Fisheries Wildlife and Conservation Biology, University of Minnesota, St. Paul, MN USA; 2 Department of Fisheries, Wildlife and Conservation Biology, College of Food, Agriculture and Natural Resource Sciences, University of Minnesota, St. Paul, MN USA; 3 Department of Veterinary Population Medicine and Veterinary Diagnostic Laboratory, College of Veterinary Medicine, University of Minnesota, St. Paul, MN USA

**Keywords:** Microbiology, Ecology, Diseases

## Abstract

Cyprinid herpesvirus-3 (CyHV-3, syn. koi herpesvirus) is an important pathogen worldwide and a common cause of mass mortality events of wild common carp (*Cyprinus carpio*) in North America, however, reference strains and genomes obtained from wild carp are not available. Additionally, it is unclear if fishes in North America are susceptible to CyHV-3 infection due to incomplete susceptibility testing. Here we present the first North American type strain and whole-genome sequence of CyHV-3 isolated from wild carp collected from a lake with a history and recent incidence of carp mortality. Additionally, the strain was used in an in-vivo infection model to test the susceptibility of a common native minnow (*Pimephales promelas*) and goldfish (*Carrasius auratus*) which is invasive in North America. Detection of CyHV-3 DNA was confirmed in the tissues of a single fathead minnow but the same tissues were negative for CyHV-3 mRNA and samples from exposed fathead minnows were negative on cell culture. There was no detection of CyHV-3 DNA or mRNA in goldfish throughout the experiment. CyHV-3 DNA in carp tissues was reproducibly accompanied by the detection of CyHV-3 mRNA and isolation on cell culture. Additionally, environmental CyHV-3 DNA was detected on all tank filters during the study. These findings suggest that fathead minnows and goldfish are not susceptible to CyHV-3 infection and that detection of CyHV-3 DNA alone in host susceptibility trials should be interpreted with caution.

## Introduction

Cyprinid herpesvirus 3, (CyHV-3, syn. koi herpesvirus), is a viral pathogen of common carp (*Cyprinus carpio*), hereafter referred to as carp, and its ornamental variety, koi carp*.* CyHV-3 was first identified in the USA in domestic ornamental koi^1^ and has since been detected with PCR-based methods in association with widespread mass mortality events of wild carp in the USA^2,3^, as well as in some clinically healthy populations^4,5^. However, isolation of CyHV-3 endemic to carp in North America has been largely unsuccessful despite the use of known permissible cell lines (koi fin, KF-1, and common carp brain, CCB) as well as other routinely used fish cell lines^3,6^. Isolation of CyHV-3 has only been reported once in 2006, and on an atypical cell line, fathead minnow cells (*Pimpephales promelas,* FHM)^7^. Since then, formation of cytopathic effect (CPE) on FHM using CyHV-3 positive tissue has not been replicated^1,2,6,8,9^. Isolation of CyHV-3 on cell lines is a critical step in establishing a causal relationship between CyHV-3 detection and disease outbreaks. Furthermore, there is a lack of study isolates for evaluating the genetic background, pathogenicity and host range of strains endemic to North America.

Though carp and its hybrids (carp x goldfish) are the only species known to be affected by koi herpesvirus disease (KHVD), the range of species which may be susceptible to CyHV-3 infections remains unclear^10^. Goldfish (*Carrasius auratus*) have been shown to become infected and able to transmit CyHV-3 to naive carp^11,12^; however, evidence of susceptibility of goldfish to infection with CyHV-3, and the ability of goldfish to act as a carrier for CyHV-3 has not been replicated in other studies^13,14^. Additionally, detection of CyHV-3 DNA and transmission to carp by cohabitation experiments has been reported in a wide variety of non-carp species from survey or disease trial studies without the presence of clinical signs^15^. In total, CyHV-3 DNA has been detected in seven fish species native to North America: rainbow trout (*Oncorhynchus mykiss*)^16^, brown bullhead (*Ameirus nebulosus*) and three-spined stickleback (*Gasterostues aculeatus*)^17^, black bullhead (*Ictaluris melas*)^18^, silver perch (*Bairdiella chrysoura*)^19^, Atlantic sturgeon (*Acipenser oxyrinchus*)^20^, and northern pike (*Esox Lucius*)^21^. The absence of clinical signs or the employment of tools for differentiating sources of CyHV-3 DNA (e.g. subclinical viral infection, environmental contamination, latent infection, or false positive) makes the interpretation of these findings difficult.

A variety of diagnostic tools are available for determining the presence of CyHV-3 in suspected cases^10^. Isolation of CyHV-3 on permissive cell lines is a definitive method for determining the presence of infectious CyHV-3 in tissue and environmental samples but permissible cell lines, Koi fin (KF-1) and common carp brain (CCB), are not highly susceptible to CyHV-3^21–23^ and are not considered a sensitive diagnostic tool^10^. Recently, cell lines with higher sensitivity to CyHV-3 have been reported^24^ but are not yet widely available.

PCR-based methods for detection of CyHV-3 DNA are the most sensitive diagnostic tools for CyHV-3. Of these assays, the Gilad qPCR is the most sensitive method^25^. In-situ hybridization has been used as a confirmatory method for CyHV-3 DNA in tissues^11^ and the use of immunochemical assays such as ELISA have been reported for determination of previous exposure of fish to CyHV-3^26–28^. RNA and mRNA detection assays are particularly useful in determining the host range of CyHV-3 since infectious hosts, in which viral replication must occur, can be distinguished from vectors, which may transport CyHV-3 DNA and infectious particles but without viral replication. Multiple PCR-based methods have been reported for the detection of CyHV-3 mRNA^12,29^; however, the mRNA detection test developed by Yuasa et al.^29^ does not detect CyHV-3 DNA when present and is thus preferable to assays that rely on DNAse enzyme treatment which may not completely remove DNA when concentrations are high.

Given the widespread detection of CyHV-3 in North America^2^ and the inevitable exposure of native fishes, it is critical to understand potential impacts of local CyHV-3 strains on invasive carp populations and on native fishes. Both native FHM and invasive goldfish are sympatric with carp and could occur in many of the same habitats. While the host status of goldfish with regard to CyHV-3 has been partially described^11,12^, demonstration of true susceptibility to CyHV-3 infection requires confirmation that indicates viral replication (i.e., presence of mRNA)^19,29,30^. The susceptibility status of FHM has never been evaluated, despite the only previous isolation of a North American CyHV-3 isolate on the FHM cell line^7^. Here, we further investigate the susceptibility of FHM and goldfish to CyHV-3 in accordance with criteria for determination of host susceptibility defined by the OIE (i.e., route of transmission is consistent with natural pathways for the infection, adequate identification of the pathogen, and provision of evidence indicating that pathogen presence constitutes an infection)^31^ using a strain of CyHV-3 that was associated with multiple, previously reported mass mortalities of wild carp in North America.

## Materials and methods

### Collection of wild carp from a CyHV-3-exposed population

This study was carried out in accordance with the recommendations in the Guide for the Care and Use of Laboratory Animals of the National Institutes of Health. All protocols for sampling, procedures and experimental endpoints involving live fish conducted in this study were approved by the Institutional Animal Care & Use Committee (IACUC), University of Minnesota (St. Paul, Minnesota, USA), under the approval numbers IACUC-1806-36036A and 1808-36276A. Experiments were performed in compliance with the ARRIVE guidelines on animal research^32^.

**Figure 1:**
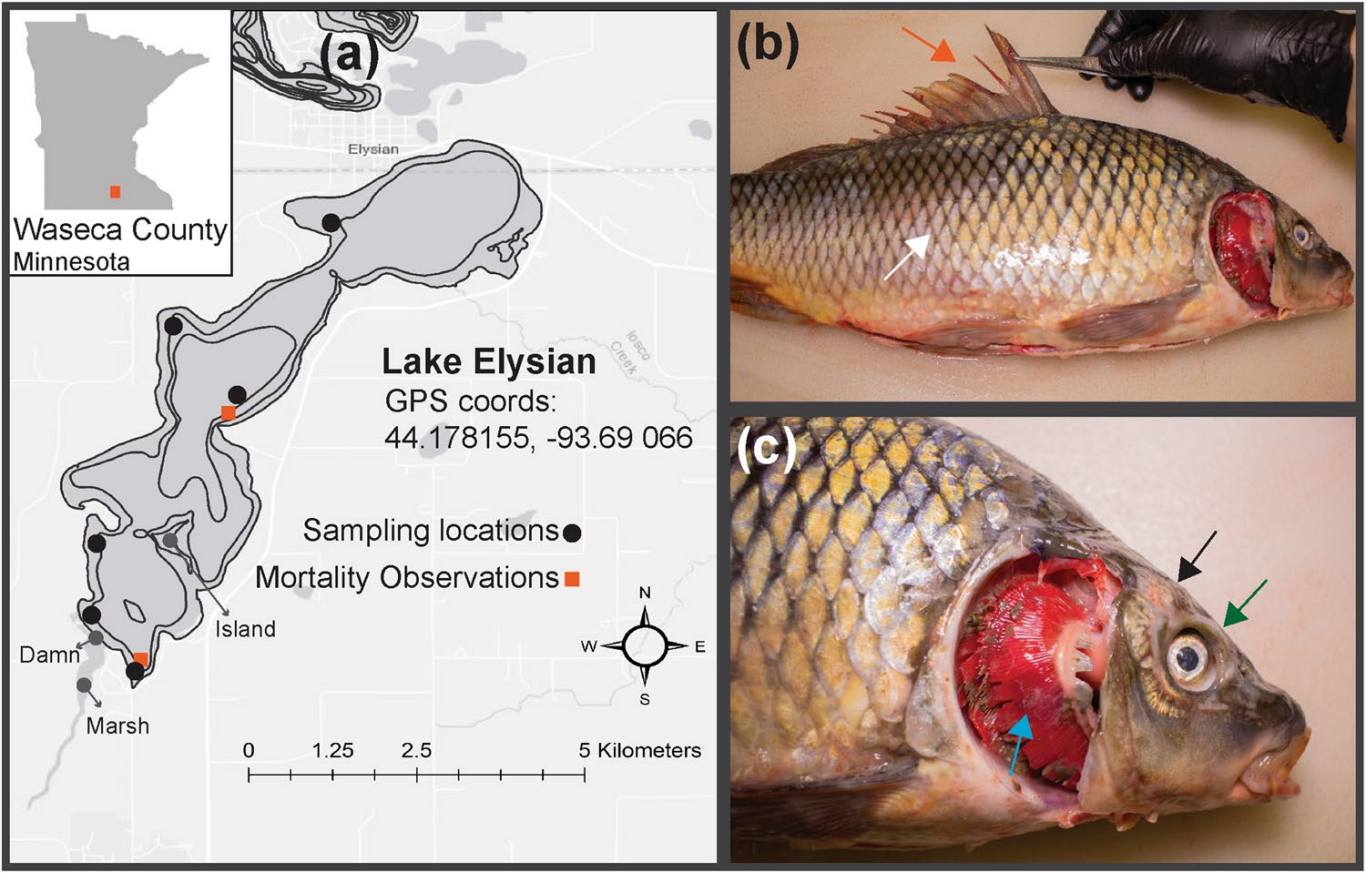
(**a**) Generated using ArcMap (v10.8.1, https://desktop.arcgis.com/en/arcmap/), shows the approximate locations of sampling effort and mortality observations on Lake Elysian. Bathymetric contours indicate depth in 5 ft increments. (**b**, **c**) Pathology of a representative individual wild carp sampled from Lake Elysian. Arrows on (**b**, **c**) denote frayed fins (vermillion), loss of mucosal layer (white), loss of scales and epidermis (black), enopthalmia (bluish green), gill necrosis (sky blue).

Wild carp were sampled from Lake Elysian (Waseca County, Minnesota, Coordinates: 44.178144, − 93.69066) by boat electrofishing from September 3 to 9, 2019 (Fig. 1a). This lake was expected to have a CyHV-3-exposed carp population following a confirmed outbreak in 2017^3^. Captured wild adult carp (n = 116) were euthanized by immersion in a solution of ~ 3 mL/L pure clove oil (90% Eugenol; Velona, Elk Grove Village, IL, USA) for 15 min and transported on ice to the University of Minnesota for necropsy. Brain, gill and kidney tissues from up to three carp were pooled in a 1:5 (weight:volume) dilution of Hank’s Balanced Salt Solution (HBSS; Cellgro, Lincoln, NE, USA) containing 100 IU/mL of Penicillin and Streptomycin and maintained at a pH of 7.4 at 4 °C for 24 h prior to preparation for qPCR and cell culture screening for CyHV-3 (described below). Gill tissues from ten freshly-dead carp obtained from a shallow bay in the Southern portion of the lake were also obtained and pooled by five individuals for a total of two sample pools.

An additional 17 wild carp collected as part of the previously described sampling event were placed in an aerated live well and transferred to the Minnesota Aquatic Invasive Species Research Center’s Containment Laboratory (MCL). These carp were housed in a ~ 1400 L tank with flow through well water at 20 °C and treated with 0.6% aquarium salt once per day. Carp were acclimated for 1 day and then anesthetized via immersion in a solution of 100 µL/L of clove oil and uniquely marked using colored injectable elastomer (Northwest Marine Technology, Anacortes, WA, USA). Additionally, a small portion (~ 0.2 cm^2^) of each carp’s gills were sampled for qPCR screening for CyHV-3 and tested immediately. Carp determined to be CyHV-3 negative (n = 12) were euthanized following testing. Carp determined to be CyHV-3-positive (n = 5) by specific qPCR were held for a total of 5 days, during which, water temperature was gradually increased to 26 °C in order to increase viral shedding. CyHV-3-positive carp gill biopsies were again sampled and screened on the fifth day to identify carp with high qPCR copy numbers. All CyHV-3-positive carp were then euthanized, and the brain, gill and kidney tissues were removed as previously described. Pooled tissues from two wild carp with clinical signs consistent with KHVD (Fig. 1b,c) and with high qPCR copy numbers, were subjected to cell culture immediately following necropsy. In addition, a 10 g portion of this pooled tissue was processed and used to challenge naive carp in the in-vivo infection model. Tissues were homogenized in a 1:5 volume of HBSS containing 100 IU/mL Penicillin and Streptomycin (pH = 7.4). The sample was centrifuged at 2360 × *g* at 25 °C for 10 min, then the supernatant was passed through a 0.45 µm syringe filter.

### In-vivo infection trial

To increase the potential of obtaining an isolate of CyHV-3, naïve carp previously determined to be CyHV-3 negative by qPCR, were challenged with CyHV-3-positive tissue homogenates obtained from wild carp. Two naïve carp, purchased from Osage Catfisheries (Osage Beach, MO, USA), were pair housed in a 60 L aquarium with flow through well water (flow rate = 3–4 tank volumes/h) at 21–22 °C. Aquaria were set up with a standpipe drain covered by a cylindrical wire screen filter of approximately 15 cm in length and 4.4 cm in diameter. Additionally, a PVC pipe section of 15 cm in length and 10 cm in diameter was added to each tank for shelter. Each carp was exposed to 0.5 mL of CyHV-3-positive tissue homogenate by IP-injection and monitored for signs of disease for 6 days and then euthanized. Pooled samples of brain, gill and kidney tissue were subjected to qPCR and cell culture analysis. Following cell culture analysis (below) a second infection trial was performed to satisfy River’s postulates (i.e. that CyHV-3 isolated from wild diseased carp would cause similar disease in naïve carp)^33^. Two additional naïve carp purchased from Osage Catfisheries were IP-injected with 0.5 mL of CyHV-3-positive (qPCR and cell culture positive) cell culture supernatant. Carp were housed and observed for disease signs as previously described for 11 days and then sacrificed. Pooled samples of brain, gill, and kidney then were tested by CyHV-3-specific qPCR to confirm the presence of CyHV-3.

### Cell culture analysis

CCB cells were maintained in Eagle’s Minimum Essential Medium (EMEM) containing Eagles’s salts (Sigma, St. Louis, MO, USA), 10% fetal bovine serum (FBS), 1% non-essential amino acids (NEAA, Sigma), 2 mM l-glutamine and glucose (Sigma) up to 4.5 g/L. The KF-1 cells were cultured in EMEM containing Eagles’s salts (Sigma), 10% FBS and 0.025 M HEPES. Penicillin 100 U/L and streptomycin 0.1 mg/L (Sigma) were used as an anti-bacterial agent in both cell culture media and the cells were maintained at 25 °C.

Cell culture methods to isolate CyHV-3 were performed according to the US Fish and Wildlife Service and American Fisheries Society-Fish Health Section Blue Book^34^. Briefly, pooled tissues were homogenized in Hank’s Balanced Salt Solution (HBSS; Cellgro) and centrifuged at 2360 × *g* for 15 min. The inoculum was added to the 24-well plates with 80% confluent cell cultures in two dilutions, (1/10 and 1/100) and incubated at 25 °C for 14 days. A blind passage was performed for an additional 14 days if no cytopathic effects (CPE) were observed on the first passage. If CPE was observed during the first passage, then the second passage was performed in a 25 cm^2^ flask. The virus was harvested when CPE reached 70–80% of the monolayer. The infected cultures were exposed to two freeze/thaw cycles at − 80 °C, and then centrifuged at 3800 × *g* for 15 min at 4 °C. The clarified supernatants and pellets were collected and stored at − 80 °C.

### Whole-genome sequencing and sequence analysis

Whole-genome sequencing was performed at the University of Minnesota Veterinary Diagnostic Laboratory for genetic characterization of the CyHV-3 isolate (KHV/Elysian/2019) obtained from wild carp. In brief, after CCB cells, infected with wild carp tissues, reached 80% CPE, the supernatant was collected and stored at − 80 °C. The frozen supernatant was freeze-thawed three times, and centrifuged at 2896 × *g* for 25 min at 4 °C. Nucleic acid purification of CCB cell culture supernatant was done using a QIAamp MinElute Virus Spin Kit (Qiagen, Hilden, Germany) following manufacturer instructions. The extracted nucleic acids were subjected to library preparation using Nextera Flex DNA library kit (Illumina, San Diego, CA, USA) following manufacturer instructions. The library was normalized according to the median fragment size measured by Tape Station 2.0 (Agilent, Santa Clara, CA, USA) and library concentration measured by Qubit. The library was submitted to the University of Minnesota Genomic Center (UMGC) for sequencing via MiSeq V3 (2X75-bp) paired end chemistry.

Raw fastq files were trimmed to remove Illumina adapters using Trimmomatic (v 0.39) with a minimum quality score of 20. Then, bowtie2 (v 2.3.5) was used to remove host contamination and unmapped reads were used for assembly with SPAdes (v3.13.0) with *k*-mer values of 29, 33 and 55 with the options “careful with a minimum coverage of 5 reads per contig”. Then contigs were searched into the RefSeq viral and non-redundant protein reference databases using Diamond BLASTx with an e-value of 1e − 5 for significant hits. Taxon assignments were made with MEGAN6 Community Edition according to the lowest-common-ancestor algorithm. ORFs prediction and genome annotation were done using Prokka (v1.14.5). The resulting alignment (GenBank accession no. MT914509) was aligned with 19 other CyHV-3 genomes available on NCBI using Mafft (v7) with the FFT-NS-2 alignment strategy and the following parameters: scoring matrix BLOUSUM62, gap open penalty 1.53, offset value 0. Model selection, maximum likelihood (ML) tree construction, and calculation of bootstrap values were done with R 4.0 (R Software) using phangorn (v2.5.5). ML trees were constructed using the top scoring model (GTR + G + I) and 100 bootstrap replicates were generated to assess the reliability of clades obtained in the tree. Additionally, this genome assembly was compared with the previously reported thymidine kinase gene sequence obtained from carp sampled during a large mortality event in Lake Elysian in 2017 (F36, GenBank accession no. MK987089).

### Investigation of species specificity

**Figure 2:**
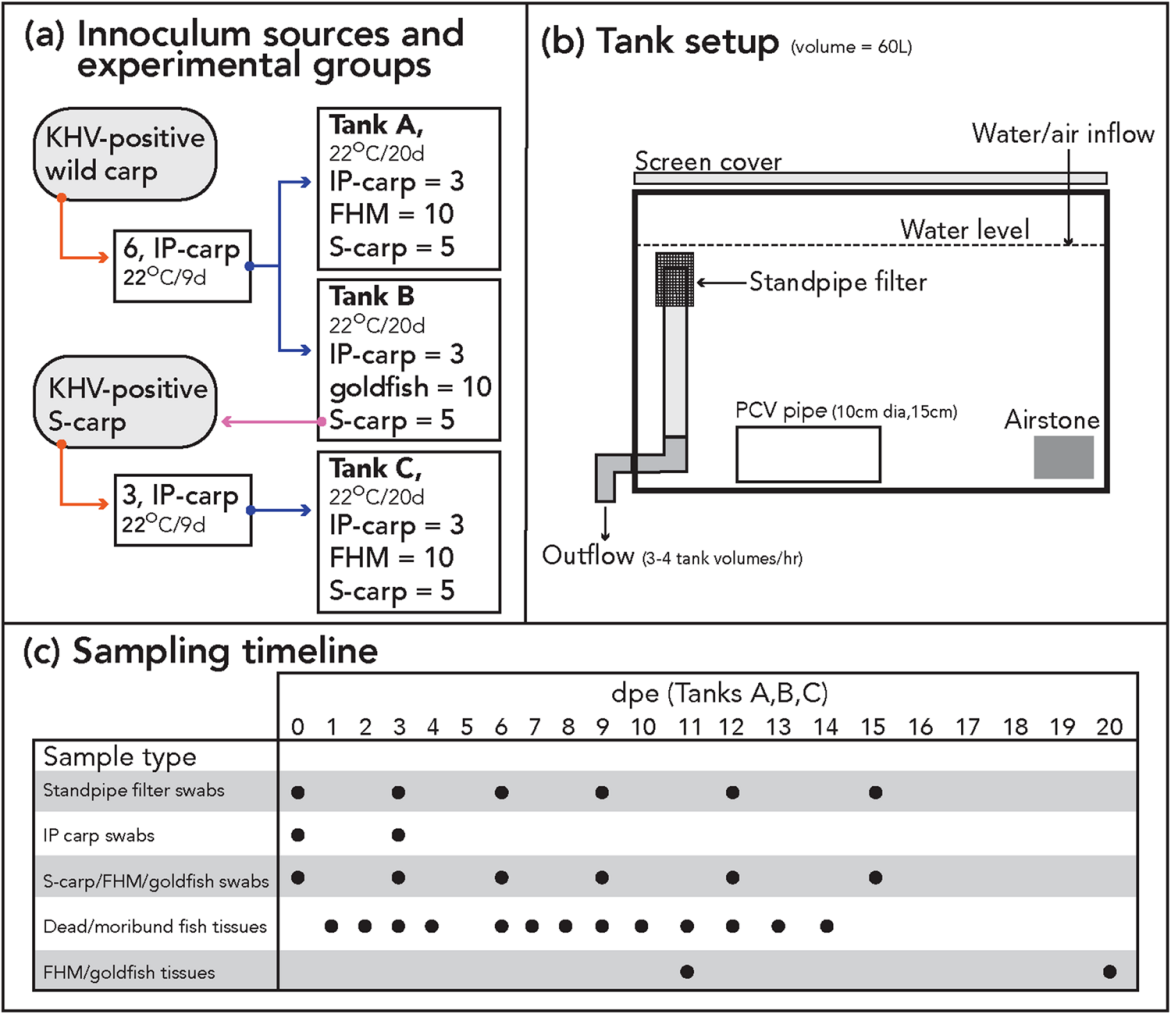
(**a**) Shows a schematic of the cohabitation disease trial. Vermillion arrows denote inoculation of IP-carp with CyHV-3 positive tissue homogenate, blue arrows denote introduction of IP carp for cohabitation with fishes in experimental tanks, and the reddish purple arrow indicates the tissue origin of CyHV-3-positive S-carp. (**b**) Shows a schematic of experimental flow through chambers with black arrows indicating the direction of water flow. (**c**) Shows a time-line of various samples.

To investigate the host range of KHV/Elysian/2019, six carp purchased from Osage Catfisheries, previously determined to be CyHV-3-negative by qPCR, were intraperitoneally (IP) injected with 0.5 mL of the filtered tissue homogenate material (Fig. 2a). The IP-injected carp (IP-carp) were housed as previously described for 9 days prior to their use in the cohabitation trial (Fig. 2b). The IP-carp were monitored twice daily for signs of disease. After 9 days the gills, skin and vent of each IP-carp was swabbed aseptically with a single sterile cotton swab (Dynarex, Orangeburg, NY, USA) for determination of viral load by qPCR. FHM and goldfish were challenged with CyHV-3 via cohabitation. One cohabitation tank (tank A) contained ten naïve FHM*,* five naïve sentinel carp (S-carp) and three IP-carp (Fig. 2a). One cohabitation tank (tank B) contained ten naïve goldfish*,* five naive S-carp and three of the IP-carp. S-carp were included in each tank setup to act as a positive control for within-tank transmission of CyHV-3. Two additional negative control tanks with the same stocking density and conditions contained ten naïve FHM (tank D) and ten naïve goldfish (tank E), as well as eight naïve carp (confirmed to be CyHV-3-negative by specific qPCR). Average standard length and weight for fishes used in these experiments was 13 cm and 64 g for carp, 7 cm and 13 g for FHM, and 10 cm and 38 g for goldfish. All tanks consisted of ~ 60 L aquaria with flow-through well water as previously described. Fishes were fed a commercial feed (Skretting classic trout, Skretting, Tooele, UT, USA) daily and monitored twice daily to observe changes to fish health. IP-carp that died during the trial were allowed to remain in the tank for 24 h prior to removal for necropsy, but any morbidity or mortality of other experimental groups were immediately removed and necropsied.

**Figure 3:**
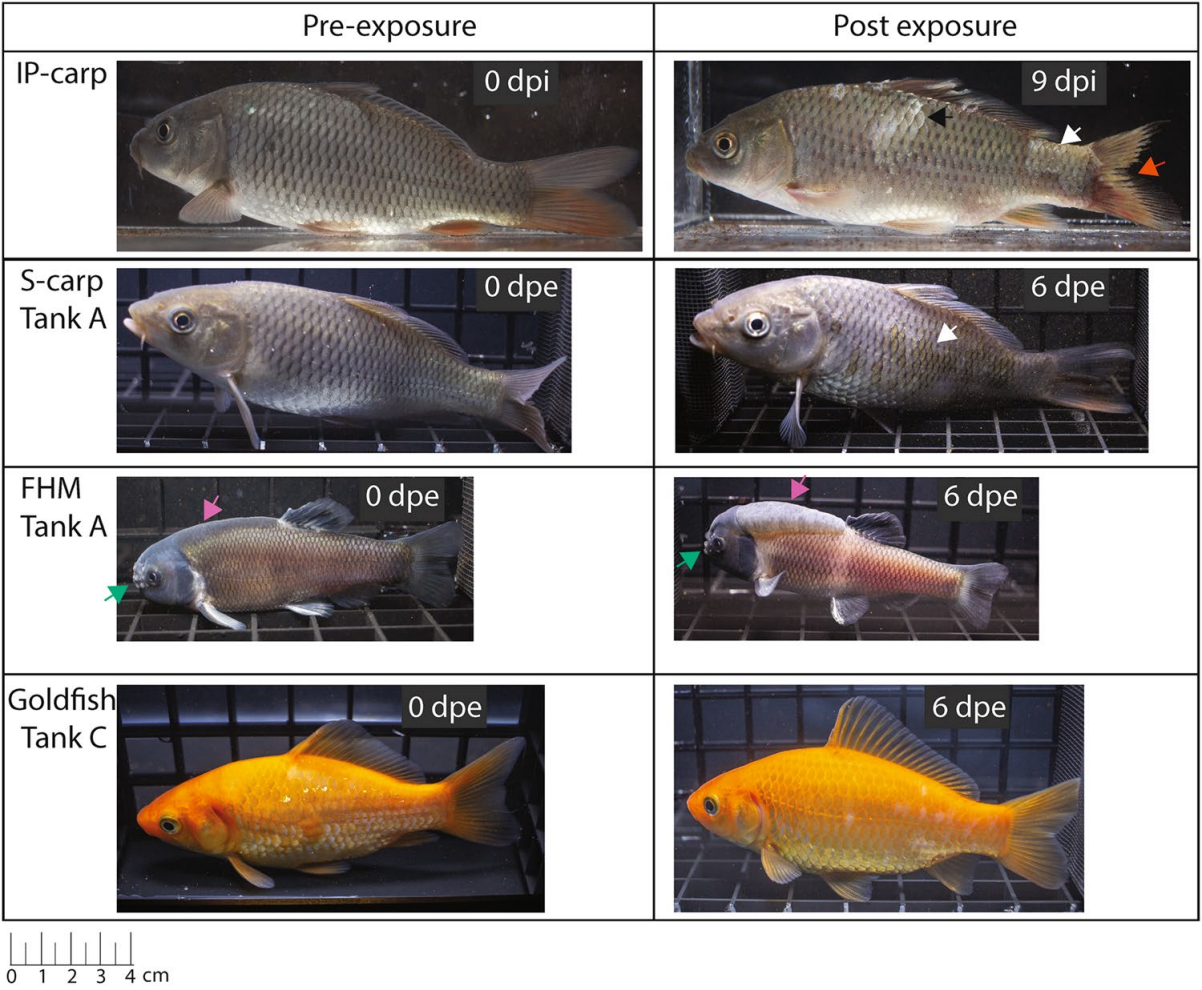
Representative fishes photographed before and after exposure to CyHV-3. Note, fishes photographed at 0 dpe may not be the same individual as those at 6 dpe. *dpe* days post exposure via cohabitation, *IP-carp* intraperitoneally injected carp, *S-carp* cohabitated sentinel carp, *FHM* fathead minnow. Arrows denote frayed fins (vermillion), loss of mucosal layer (white), scale pocket edema (black). Additionally, normal morphological features of mature male fathead minnows are indicated for nuptial tubercles (bluish green), and nape pads varying in prominence (reddish purple).

At 0, 3, 6, 9, 12, and 15 days post exposure (dpe) by cohabitation, five FHM, five goldfish, and all IP-carp and S-carp from each tank were anesthetized by immersion in a buffered solution of 100 mg/L of MS-222 and the gills, skin and vent of each fish was swabbed with a sterile swab for determination of viral load by qPCR (Fig. 2c). For FHM and goldfish, the five individuals were randomly sampled at each time-point. Additionally, the wire screen filter of the outflow standpipe was swabbed at the same intervals during the course of the trial to evaluate loading of CyHV-3 DNA in the environment. All swabs were stored at − 20 °C in individual plastic bags until nucleic acid extraction could be performed. At 11 dpe, half of the FHM and goldfish from cohabitation tanks were euthanized by immersion in a buffered solution of 3 g/L of MS-222 and necropsied (Fig. 2c). The remaining FHM and goldfish were maintained until 20 dpe and then euthanized and necropsied. To visually record the presence of gross pathology, representative IP carp, and fish from cohabitation groups (S-carp, FHM, and goldfish) were randomly selected and photographed at 0 and 6 dpe in a small glass aquarium (Fig. 3).

For each necropsied fish, wet mounts of gill and skin scrapes were viewed at 40× magnification to identify potential parasitic infections. Then the skin of each necropsied fish was rinsed briefly with 70% ETOH and clean water. Brain, gill, kidney and skin tissue were collected individually for each fish and split into two duplicate samples. The first sample duplicates were placed in Whirl–Pak sample bags (Nasco, Fort Atkinson, WI, USA) and preserved at − 20 °C until nucleic acid extraction and screening for CyHV-3 DNA was performed. The second sample duplicates were placed in 1 mL of RNA*later* solution (Ambion) in 1.5 mL microcentrifuge tubes (Globe Scientific, Mahwah, NJ, USA) and frozen at − 20 °C. An individual FHM and goldfish from each time-point (11- and 20-dpe) was preserved in 10% NBF (TissuePro, Gainesville, FL, USA) for histological analysis. Individual representatives of each species from control tanks and moribund S-carps from each experimental tank were also preserved for histological analysis.

Due to the detection of CyHV-3 DNA in a single FHM in tank A, a second trial with FHM (tank C) was performed as described previously (Fig. 2a). Brain, gill, kidney, and skin tissue from two S-carp exposed in the first trial with disease signs and positive qPCR test for CyHV-3 (tank A) were pooled, homogenized and filtered as previously described. Three new carp purchased from Osage Catfisheries were IP injected with 0.5 mL of this tissue homogenate and maintained as previously described for 9 days prior to screening for CyHV-3 by qPCR and used in the cohabitation trial. All other conditions and procedures were done as described for the first cohabitation trial with the following exceptions. In the second trial, portions of brain, gill, kidney and skin tissues obtained from a moribund S-carp at 5 dpe and four FHM at 11 dpe, respectively, were pooled as previously described and subjected to cell culture. Additionally, duplicate swabs from the tank C outflow standpipe filter were obtained and preserved in 1 mL of RNA*later* solution (Sigma) as previously described for tissue samples.

### Nucleic acid purification using chelex resin and detection of CyHV-3 by qPCR

For nucleic acid purification, chelex resin (Sigma) was used as described by Zida et al.^35^ and briefly summarized here. For pooled tissue samples, approximately 100 mg of each tissue was homogenized in 1 mL of nuclease free water (NFW) and then centrifuged, with 50 μL of the resulting supernatant later used as starting material. For swabs, the cotton end was cut off and vortexed, then centrifuged and finally the cotton was removed leaving the supernatant. For each sample type, 150 μL of chilled 80% ETOH was added, then centrifuged and the supernatant removed. Samples were allowed to air dry for 10 min to remove residual ETOH. 150 μL of 20% Chelex was added to each sample and vortexed. Samples were then incubated at 90 °C for 20 min and centrifuged and immediately used for qPCR.

A Taqman probe-based qPCR was used for the detection of CyHV-3 DNA targeting the ORF89 gene^36^ using a StepOnePlus thermocycler with default settings (Applied Biosystems). Nucleic acid purifications from all samples were screened for CyHV-3 using a PrimeTime gene expression master mix kit (Integrated DNA Technologies, Coralville, IA, USA), with each reaction containing 400 nM of primers (KHV-86f: GAC-GCC-GGA-GAC-CTT-GTG, KHV-163r: CGG-GTT-GTT-ATT-TTT-GTC-CTT-GTT) and 250 nM of the probe (KHV-109p: [TAMRA] CTT-CCT-CTG-CTC-GGC-GAG-CAC-G-[IBRQ]. The reaction mix was subjected to an initial denaturation at 95 °C for 3 min, followed by 40 cycles of denaturation at 95 °C for five sec and annealing at 60 °C for 30 s. A threshold cycle of 38 was used as a cut off. The standard curve for quantification of CyHV-3 genomes was performed using a laboratory synthesized DNA fragment containing the ORF89 sequence as previously described by Padhi et al.^3^. The results for virus load are presented as the number of viral copies per mL of tissue supernatant. All samples obtained from FHM and goldfish were tested in triplicate with the exception of samples that had positive qPCR Ct values, which were re-tested up to six times.

### RNA purification and reverse transcription polymerase chain reaction (RT-PCR)

Individual tissues of preserved brain, gill, kidney, and skin from one representative S-carp from each experimental tank (A, B and C) were selected as positive controls for CyHV-3 mRNA detection (total of 12 tissue samples). All preserved tissue samples from FHM or goldfish which had at least one positive qPCR test were also screened for CyHV-3 mRNA to determine if replicating virus was present (total of eight tissue samples). Additionally, preserved swabs of the outflow standpipe filter were also screened. For RNA purification, RNA was extracted from tissues using the RNeasy Mini Kit (Qiagen) according to the manufacturer instructions for animal tissues, using ~ 30 mg tissue samples preserved in RNA*later*. For swabs, cotton was cut from the end of the swab and used as the starting material. CyHV-3 mRNA was detected using the RT-PCR developed by Yuasa et al.^29^ with the primers, (KHV RT F3: GCC-ATC-GAC-ATC-ATG-GTG-CA, KHV RT R1: AAT-GCC-GCT-GGA-AGC-CAG-GT). The RT-PCR was performed using a One-step RT-PCR kit (Qiagen) according to the manufacturer instructions. The reaction mix was subjected to a single step of reverse transcription at 50 °C for 30 min and denaturation at 95 °C for 15 min, followed by 40 cycles of: 94 °C for 30 s, 65 °C for 30 s, 72 °C for one minute and a final extension step was 72 °C for 10 min. PCR products were separated and visualized on 2% agarose gels containing 0.75 μg/mL ethidium bromide (Genesee Scientific, San Diego, CA, USA). PCR products for carp, FHM and goldfish templates (clear band at the 219 bp location) were cut from gels and purified by precipitation with a 20% PEG, 2.5 M NaCl solution. Purified RT-PCR products were subjected to Sanger sequencing at the University of Minnesota Genomics Center (UMGC). Sequences were trimmed and analyzed using 4 peaks (v1.8) and consensus sequences were generated using BioEdit (v7.2.1). Sequence identities were compared with available reference sequences by BLASTn analysis of the National Center of Biotechnology sequence database.

### Histology

Histology was used to demonstrate the presence or absence of lesions in cohabitation disease trial specimens. Histological samples of gill tissue were prepared from formalin-fixed samples of representative fishes of each species from trial and control tanks. Gill samples were dissected from formalin-fixed specimens and decalcified in 10% ethylenediaminetetraacetic acid (EDTA) for 10 days. Following decalcification, samples were dehydrated in an ethanol series to 100% ethanol, infiltrated with toluene, and subsequently embedded in paraffin. Paraffin sections were cut at 6 µm thickness using a Leica Jung 820 Histocut Rotary Microtome and mounted on slides. Sections were stained with Hematoxylin and Eosin using a protocol modified from Humasson^37^.

### Statistical analysis

R 4.0 (R Software) was used for statistical analysis and data presentation. CyHV-3 qPCR copy numbers are presented as averages of all positive tests for samples with duplicate tests and were Log transformed prior to statistical testing. Significant differences (*p* < 0.05) in virus load of IP injected carp and cohabitated fish were determined using a 1-way ANOVA with subsequent pairwise multiple comparisons using the Holm-Sidak method and data were presented as box plots of 25–75% (+ minimum and maximum values) with an indication of mean and median. Bivariate associations were measured with odds ratios and 95% confidence intervals (Cis).

## Results

### Detection and isolation of CyHV-3 from wild carp

Wild carp obtained from Lake Elysian (n = 133; 116 immediately euthanized and 17 transferred live to the MCL) ranged in standard length from 10 to 68 cm (mean standard length = 49.16 cm, SD = 10.03 cm) and in weight from 0.95 to 6.29 kg (mean weight = 2.94 kg, SD = 1.42 kg). Among this sample of wild carp 36 were male, 93 were female, and four were young of the year (sex could not be determined). Water temperature at the time of sampling was between 20 and 22 °C at the surface.

Pooling of tissues from wild carp which were immediately euthanized for screening (n = 116) resulted in a total of 50 tissue pools. A total of 10/50 tissue pools tested positive for CyHV-3 by qPCR with copy numbers ranging between 2.81E + 03 and 1.63E + 06 (mean copy no. = 2.53E + 05). Gill necrosis was observed in 18 wild carp (15.52%) and positively associated with the detection of CyHV-3 DNA by qPCR (p-value = 8.20E − 07, OR = 89.37, 95% CI 8.60 4.78E + 03). However, the presence of gill necrosis was not observed in all fish with positive qPCR tests. Two gill tissue pools collected from dead carp also tested positive for CyHV-3 by qPCR with copy numbers of 6.03E + 05 and 2.36E + 06.

Five of the 17 live carp tested positive for CyHV-3 in gill samples with copy numbers ranging from 8.03E + 03 to 4.18E + 05. The two carp with the highest qPCR copy numbers of 4.18E + 05 and 2.04E + 04 were used for cell culture inoculum and the in-vivo infection trial. Pooled tissue homogenate from these fish used in disease trials and cell culture had a copy number of 2.00E + 04. The two live wild carp that had the highest qPCR copy numbers for CyHV-3 also had signs of disease including necrotic gills, frayed fins, apparent loss of the mucosal layer (i.e. rough sandpaper-textured scales), loss of scales and epidermis, and enopthalmia (Fig. 1b,c). *Gyrodactylus sp.*, *Trichodina sp.* and *Flavobacterium columnare* were also observed in wet mounts of skin and gill scrapes from several of the wild carp housed in the MCL. There were no clinical signs present in the other 15 carp held in the MCL, including three additional CyHV-3 positive carp and 12 that were negative.

Tissue pools (n = 50) from 116 wild carp used for cell culture inoculum did not produce CPE on KF-1 or CCB cell lines. However, CPE was observed on both the KF-1 and CCB cell lines when inoculated with tissue of the carp held in the MCL. The CPE was characterized by the detachment of cells from the surface of the substrate along with extensive vacuolation 9 days post-inoculation of the blind passage. The cultures were confirmed to be CyHV-3-positive by qPCR with a viral copy number of 2.02E + 08.

### In-vivo infection trial

River’s postulates for associating CyHV-3 with disease of wild carp in Lake Elysian were fulfilled in the initial in-vivo infection trial. Disease signs and subsequent morbidity at 6 dpe were observed in naïve carp injected with CyHV-3-positive tissues obtained from the wild carp. Pooled samples of brain, gill, and kidney obtained from experimentally infected individuals were positive for CyHV-3 with a qPCR copy number of 2.60E + 06 and produced CPE on CCB cells 14 days after inoculation. This CPE-positive CCB supernatant had a qPCR copy number of 5.89E + 06. Additional naïve carp injected with this cell culture supernatant were determined to be moribund at 11 dpe and were positive for CyHV-3 with a qPCR copy number of 9.74E + 05.

### Whole-genome of CyHV-3 from wild carp

**Figure 4:**
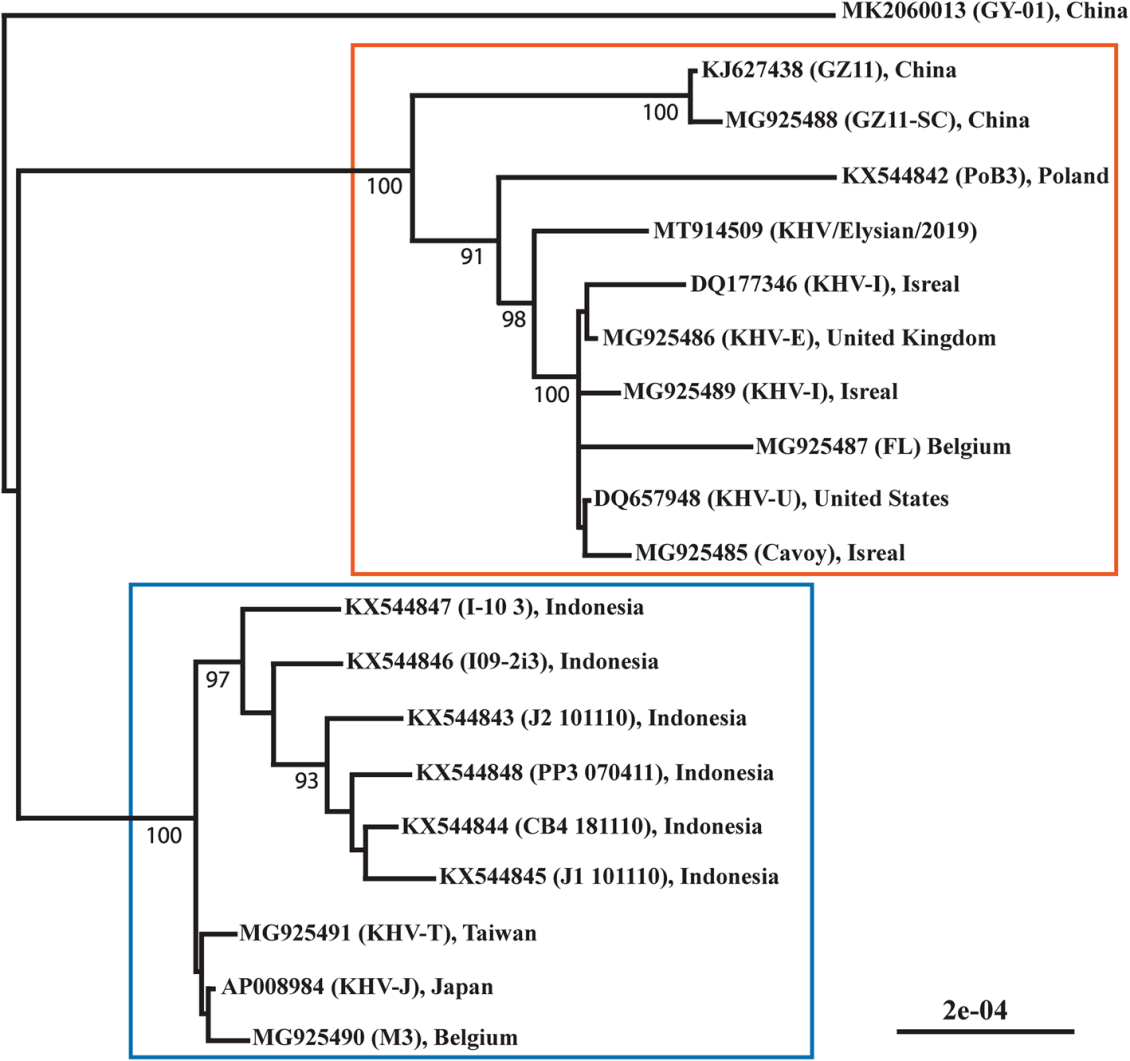
Midpoint-rooted maximum likelihood phylogeny of publicly available CyHV-3 genomes and KHV/Elysian/2019. The scale bar represents substitutions per site. Genotypes are indicated by vermillion (European) and blue (Asian) boxes.

Sequencing and denovo assembly of the KHV/Elysian/2019 genome resulted in a 295,161 bp sequence. A total of 288 parsimoniously informative sites were identified among the 20 strains included in the genome alignment. KHV/Elysian/2019 clustered with genomes in the European clade, sharing between 98.74 and 99.16% sequence identity with genomes of the Asian lineage and 99.29–99.60% sequence identity with genomes of the European lineage (Fig. 4). KHV/Elysian/2019 was most identical to KHV-E (ACC no. MG925489) which was isolated from England and first reported by Klafack et al.^38^. The previously reported 1003 bp sequence containing the thymidine kinase gene obtained from dying carp during a large mortality event in Lake Elysian in 2017 (CyHV-F36, ACC no. MK987089) was identical to that of KHV/Elysian/2019.

### Susceptibility of carp, fathead minnow and goldfish to a North American strain of CyHV-3

**Figure 5:**
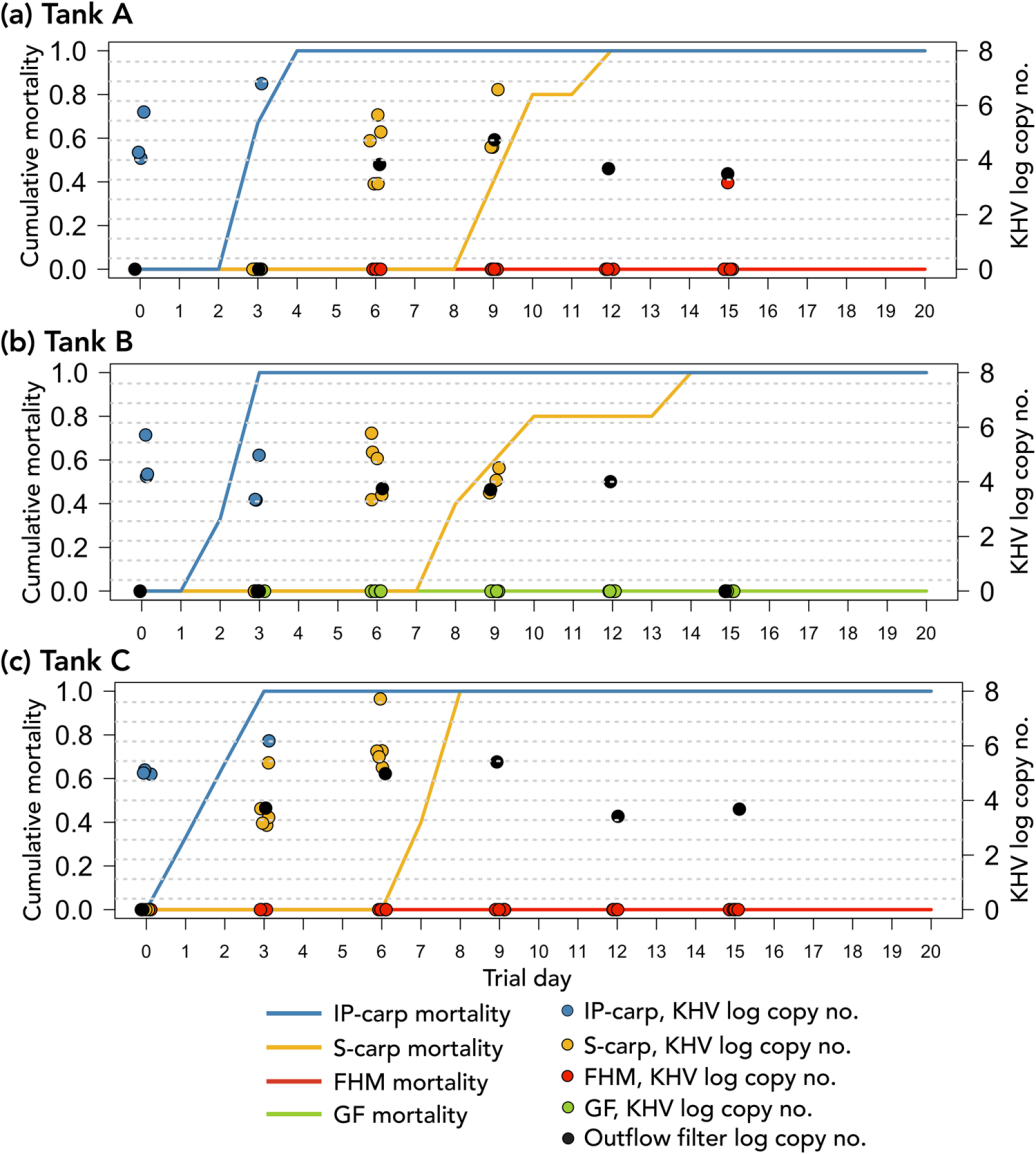
(**a**–**c**) The cumulative mortality and CyHV-3 log copy number/mL of fishes in experimental cohabitation tanks. *IP-carp* intraperitoneally injected carp, *S-carp* cohabitated sentinel carp, *FHM* fathead minnow, goldfish. “Trial day” indicates days post introduction of IP carp into cohabitation tanks. Points indicating log copy number are jittered to aid in visualization.

After 4–6 days post IP injection with CyHV-3-positive tissue obtained from wild carp IP-carp began to show signs of disease. Clinical signs included a lack of response to feed, loss of the mucosal layer, frayed fins, pale and discolored skin, enopthalmia and lethargy (Fig. 3). IP-carp in all experimental tanks died between 9 and 12 days post injection (within 4 days of initiation of the cohabitation trial) (Fig. 5). S-carp in tanks A, B and C began showing similar signs of disease as IP-carp after 6 days (Fig. 3). Between 7 and 12 dpe, disease signs in IP- and S-carp became more pronounced and included lethargy, loss of scales and epidermis, scale pocket edema, enopthalmia and diffuse hemorrhages of the skin. Gill erosions were also observed in some IP-carp and S-carp during necropsy. No external parasites were observed on wet mounts from any individuals from experimental or control tanks.

The cumulative mortality of all IP and S-carp was 100%, occurring between 7 and 13 days after exposure to CyHV-3 inoculum or infected carp (Fig. 5). Onset of mortality and complete mortality of naive carp occurred earlier in tank C than in tank A. No lesions or change in behavior of FHM or goldfish were observed in any of the experimental tanks (Fig. 3). However, a single mortality of FHM occurred in the negative control tank (tank D); but no lesions were observed during necropsy.

**Figure 6:**
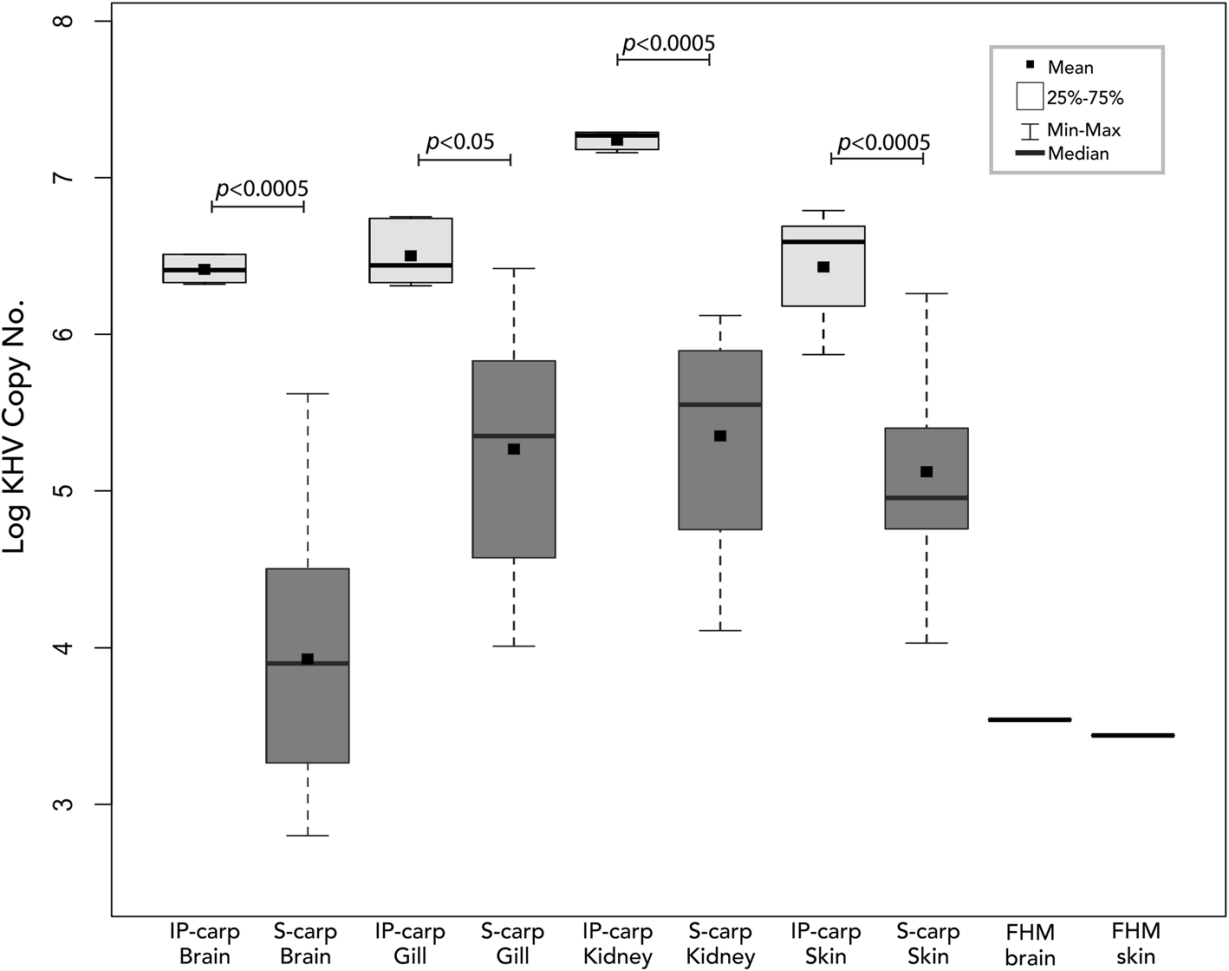
Boxplots of CyHV-3 log copy numbers/mL of tissue supernatant of IP injected carp (IP-carp, light grey boxes), cohabitated sentinel carp (S-carp, dark grey boxes), and in tissues of a single fathead minnow (FHM), quantified using CyHV-3-specific qPCR. No boxes are displayed for FHM tissues since only a single individual tested positive for CyHV-3.

The pooled tissue obtained from wild carp used for IP injection of IP-carp in tanks A and B had a copy number of 1.13 E + 04. The pooled tissue obtained from tank A S-carp, used for IP injection of IP-carp in tank C, had a copy number of 7.97 E + 05. All swabs and tissues obtained from IP-carp tested positive for CyHV-3 DNA at day 0 of the cohabitation trial (Fig. 5). Swabs from S-carp began to test positive for CyHV-3 at 3 dpe in tank C and at day 6 in tanks A and B. Swabs from all S-carp continued to test positive until none remained in cohabitation tanks. All tissues of IP- and S-carp tested positive for CyHV-3 DNA (Fig. 5). Copy numbers were significantly higher in kidney tissue from IP-carp than tissues from cohabitated carp. On average, all tissues from IP-carp had higher copy numbers than tissues from cohabitated fish (Fig. 6). Tissues from tank C S-carp were also used to re-isolate CyHV-3 on CCB cells. CPE was observed in the first passage (14 dpe) after inoculation with S-carp tissues collected from tank C.

Swabs from the outflow standpipe filter of all CyHV-3-exposed tanks were positive for CyHV-3 DNA with copy numbers between 3.12 E + 03 and 5.50 E + 04, beginning at day 3 in tank C and at day 6 in tanks A and B and continuing until day 12 in tank B and day 15 (final day of sampling from outflow standpipe filter) in tanks A and C.

A skin swab from a single FHM at 15 dpe tested positive for CyHV-3 DNA (2/3 tests positive) with an average copy number of 1.48 E + 03. All other swabs from FHM and goldfish were negative for CyHV-3 DNA. CyHV-3 DNA was detected (4/6 tests positive) in a single skin tissue sample from FHM collected on day 11 from tank A with an average copy number of 2.74 E + 03, as well as in brain from the same individual (2/3 tests) with an average copy number of 3.45 E + 03. No other tissues from FHM collected on day 11 from tank C were positive for CyHV-3 DNA. One goldfish from tank B tested positive for CyHV-3 in brain tissue (1/6 tests positive) with a copy number of 4.59 E + 03. A second goldfish in tank B tested positive for CyHV-3 in gill tissue (1/6 tests positive) with a copy number of 2.64 E + 03. No other tissues from goldfish tested positive for CyHV-3 DNA on day 11 dpe and no tissues from FHM or goldfish tested positive for CyHV-3 DNA on day 20 dpe. Tissues from all fishes in negative control tanks (tanks d and e) were all negative for CyHV-3 at 20 dpe. Additionally, tissues from FHM from tank C collected at 11 dpe and subjected to cell culture analysis did not produce CPE on CCB cells during the first or second passages.

**Figure 7:**
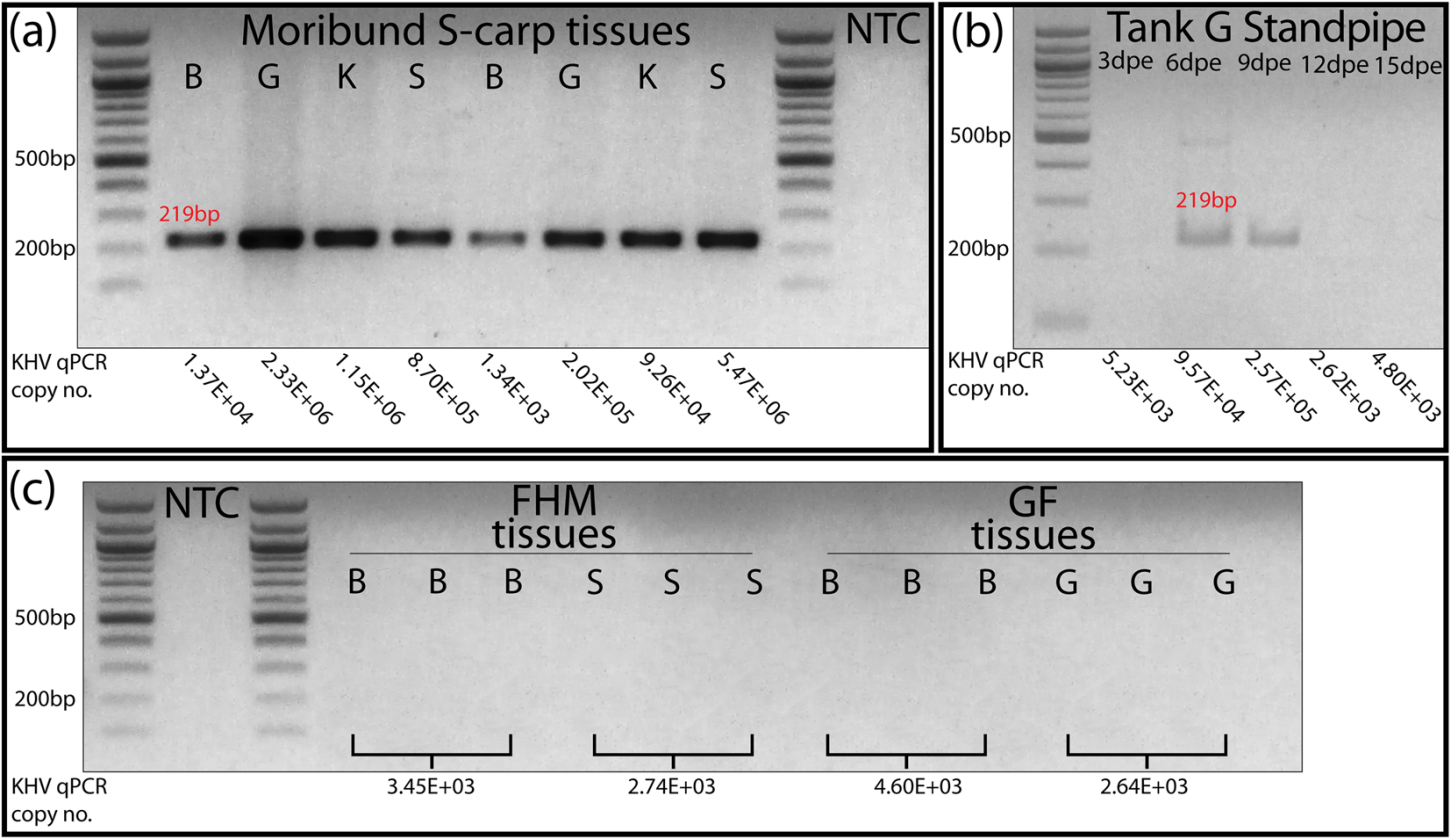
(**a**–**c**) Show UV fluorescence of RT-PCR products on 2% agarose gels stained with ethidium bromide. Bands for 500 and 200 bp on the DNA ladders are noted. The position of 219 bp is denoted in red. *S-carp* cohabitated sentinel carp, *FHM* fathead minnow, goldfish, *B* brain, *G* gill, *K* kidney, *S* skin, *NTC* no template control. In (**b**) “standpipe” indicates swabs of the tank outflow standpipe filer. CyHV-3 specific qPCR copy numbers/mL of tissue supernatant from corresponding tissues are displayed below each lane. Samples in (**a**) are representative tissues from S-carp from tank A (first 4 samples) and from tank C (last 4 samples). Samples in (**c**) are triplicate tests of FHM and goldfish tissues which had positive qPCR tests.

All tests of tissues from representative S-carp were positive for CyHV-3 mRNA with bands at the expected size of 219 bp (12 tissue samples run in duplicate) (Fig. 7). None of the tissues from FHM or goldfish were positive for CyHV-3 mRNA (eight tissue samples run in triplicate). Additionally, outflow standpipe filter swabs from tank C at days 6 and 9 of the trial were also positive for CyHV-3 mRNA. CyHV-3 specific qPCR copy numbers for corresponding tissues from S-carp testing positive for CyHV-3 mRNA ranged between 1.34E + 03 and 5.47E + 06. CyHV-3 specific qPCR copy numbers for corresponding outflow standpipe filter swabs were 9.57E + 04 at 6 dpe and 2.57E + 05 at 9 dpe (Fig. 7).

Sanger sequencing of 219 bp RT-PCR products from CyHV-3 infected carp confirmed the identity of these fragments as the CyHV-3 DNA packaging terminase subunit gene sequence (ORF33 of the CyHV-3 E genome). Sanger sequencing of purified gel extracts cut from the approximate location of potential low amplification RT-PCR products from FHM and goldfish tissues resulted in no visible reaction.

Representative fish from each treatment group were examined by light microscopy. There were no lesions in the FHM, goldfish or control carp. However, lesions consistent with KHVD were observed in the gill tissue of CyHV-3-exposed carp, including fusion of the secondary lamellae to erosion of the primary lamellae, as well as sporadic intranuclear inclusion bodies within epithelial cells.

## Discussion

The primary goal of this study was to isolate a virulent strain of CyHV-3 present in wild carp in North America and evaluate the susceptibility of two species, FHM and goldfish using a combination of methods chosen to confirm the presence of viable CyHV-3 infection. Of these two species goldfish have been previously tested for susceptibility to CyHV-3, but interpretation of results has been contradictory^11,12,14^. Under the experimental conditions in the present trial, our results indicate that FHM and goldfish are unlikely to be natural hosts for CyHV-3 and are not susceptible to KHVD. We recommend this diagnostic framework be adopted for future CyHV-3 susceptibility studies.

This is the first reported isolation of CyHV-3 in North America that has been linked with a mortality event of wild carp and fulfilled Koch’s postulates. Successful isolation of CyHV-3 had previously been documented by Grimmet et al.^7^, however, this isolate could not be maintained in cell culture, was not re-exposed to carp and only the molecular sequence of the TK gene is available. The difficulty of culturing CyHV-3 is most likely due to relative fragility of virions, limited number and susceptibility of available cell lines^8^. In this study, none of the samples of CyHV-3 positive carp tissues obtained from wild carp and maintained for 24–48 h at 4 °C induced formation of CPE. However, CyHV-3 was successfully isolated when standard isolation protocols were used in concert with live housing of infected carp and the in-vivo infection model to ensure that CyHV-3-positive tissues were subjected to cell culture shortly (less than 2 h) after necropsy. This method was also useful since carp could be screened non-lethally and euthanized during the peak of infection in order to avoid expending time and resources on negative samples or samples with low viral loads. These results reinforce the necessity of using either very fresh tissues from wild carp or tissues from carp with severe acute infections for cell culture isolation. Inoculation of disease-free carp with CyHV-3 positive tissue homogenate may be a useful preliminary step in propagating CyHV-3 in-vivo if cell culture is not immediately available.

Despite the importance of CyHV-3 worldwide, there are few publicly available genomes. Furthermore, most CyHV-3 genomes available originated from cultured carp and koi and thus, CyHV-3 genomes originating from wild carp are especially limited. The genome sequence of the isolate obtained in this study is of the same lineage (European) as that of CyHV-3 detected in Elysian in 2017^3^, as well as most strains of CyHV-3 originating prior to 1999 when Asian lineage strains began to emerge in the USA^39^. Anthropogenic movement of wild carp in the USA has been under strict regulation since the 1980s and it was well documented that carp was introduced to North America from Europe, much earlier, in the late 1800s^40^. Based on the similarity of the genome presented here to other European isolates it can be speculated that CyHV-3 in North America may have been circulating in wild populations prior to its initial report in 1998^1^. This hypothesis however should be tested by further molecular epidemiological studies.

CyHV-3 DNA was detected in at least one replicate qPCR test in brain and skin tissue of a single FHM and brain and gill of a single goldfish but is unlikely to indicate the presence of replicating CyHV-3 as evidenced by the lack CyHV-3 mRNA detection by RT-PCR. In contrast and as expected, all tissue samples from S-carp were positive for CyHV-3 DNA and mRNA, which was confirmed by Sanger sequencing and histopathology. An alternative interpretation of the DNA-positive and mRNA-negative results for FHM and goldfish may be due to a higher sensitivity of the qPCR assay and the presence of very low-level infections that are not detectable by the mRNA assay. However, this explanation is unlikely since S-carp tissues with lower CyHV-3 DNA concentrations than those of positive FHM and goldfish samples had detectable levels of mRNA. Yuasa et al.^29^ also demonstrated the sensitivity of the Yuasa RT-PCR assay to detect CyHV-3 mRNA in highly diluted positive control samples. Additionally, re-isolation of CyHV-3 on CCB cell culture from S-carp tissues was successful though CyHV-3 could not be re-isolated in cell culture from tissues of cohabitated FHM and goldfish. Finally, replication of the FHM disease trial (tank C) did not result in additional positive qPCR tests of any FHM swabs or tissues. Taken together, these results indicate that detection of CyHV-3 DNA in FHM and goldfish tissues likely originated from non-viable CyHV-3 virions or naked DNA.

The source of CyHV-3 DNA in external tissues of FHM (skin) and goldfish (skin and gill) was likely due to contact contamination with feces, mucous and sloughing skin of infected cohabitated carp. Similar fomites were also likely the origin of CyHV-3 DNA on the outflow standpipe filter, which was detectable by qPCR at least 6 days after all S-carp had died and were removed from the tank. Interestingly, CyHV-3 mRNA was also detectable on the outflow standpipe though it appeared to be partially degraded based on the appearance of faint bands. The likely source of this mRNA was carp epidermal cells that became entrained in the filter mesh of the outflow standpipe.

Given our inability to detect CyHV-3 in tissues of all but one FHM in tank A and none in the replicate trial in tank C, the source of CyHV-3 DNA in an internal tissue of an FHM (brain) is most likely the result of contamination of this sample with CyHV-3 virions or DNA on the skin or in oral cavity contents of the same individual during necropsy. However, it is also possible that this DNA originated from CyHV-3 virions which invaded or were taken up by FHM cells but without evidence of replication. CyHV-3 DNA has been previously detected in internal tissues of various fish species without evidence of replication or disease^41^. Several species of fish have also been shown to transmit CyHV-3 to naive carp under experimental cohabitation conditions, despite no detectable replication^11,13,21^. Thus, it has been speculated that many fish species may act as carriers of CyHV-3, without the presence of disease signs or detectable mRNA^16^. The application of mRNA detection assays could be further improved by development of a quantitative assay, which would be useful to verify the results of species which are listed as having incomplete evidence for susceptibility^31^, particularly if detection of CyHV-3 DNA represents sample contamination.

The difficulty in discriminating viable and non-viable aquatic pathogens (i.e. in non-viable virions or naked DNA) in environmental sources and animal tissues is a well-known limitation of molecular tests^42–44^. This is a particular challenge for CyHV-3, where molecular tests are the only reliable methods of detection^10^. The availability of a validated mRNA detection test is a convenient secondary test for confirmation of replicating CyHV-3 infections, though this test has only been employed in non-carp species susceptibility testing in two recent studies. For example, using the Gilad-qPCR and Yuasa-RT-PCR, McColl et al.^19^ evaluated the species-susceptibility of a variety of species and found no detections of CyHV-3 mRNA despite an unexpectedly high detection rate of CyHV-3 DNA. Though not conclusive, the authors interpreted this discrepancy as a high likelihood of contamination occurring during sample collection and processing. In addition, Kim et al.^30^ used the Yuasa-RT-PCR to confirm the resistance of Crucian carp (*Carrasius auratus langsdorfii*) to CyHV-3, but the authors did not accurately report the mRNA primers. No other studies acknowledge the potential for false-positive detections of CyHV-3 DNA in susceptibility trials—an oversite considering the high sensitivity of PCR-based detection methods and possibility for non-viable virions or sample contamination^45^.

Despite reduced sources of free CyHV-3 DNA contamination in this study (i.e. use of a flow through system, few within-tank structures for fomite accumulation, and washing fish exteriors) it is likely that false-positive detection of CyHV-3 still occurred in FHM and goldfish tissue samples. Unlike previous studies, we tested environmental samples (tank outflow standpipe filter) to confirm this hypothesis. Indeed, CyHV-3 DNA concentrations in the FHM and goldfish tissues samples were generally lower than those from the tank standpipe, even 6 days after all carp were removed. These findings highlight the fact that efforts should be made to minimize contamination risk during the study design and sample collection. In addition, this suggests qPCR results should be interpreted with caution, in particular when complimentary RT-PCR is not used for confirmation.

## Data Availability

The datasets generated during and/or analyzed during the current study are available in the Data Repository for the University of Minnesota, (https://conservancy.umn.edu/handle/11299/216462). Sequence data is available at the NCBI database (ACC no. MT914509).
